# Fluorinated Cannabidiol Derivatives: Enhancement of Activity in Mice Models Predictive of Anxiolytic, Antidepressant and Antipsychotic Effects

**DOI:** 10.1371/journal.pone.0158779

**Published:** 2016-07-14

**Authors:** Aviva Breuer, Christeene G. Haj, Manoela V. Fogaça, Felipe V. Gomes, Nicole Rodrigues Silva, João Francisco Pedrazzi, Elaine A. Del Bel, Jaime C. Hallak, José A. Crippa, Antonio W. Zuardi, Raphael Mechoulam, Francisco S. Guimarães

**Affiliations:** 1 Institute for Drug Research, Medical Faculty, Hebrew University, Jerusalem, Israel; 2 Departments of Pharmacology, Medical School of Ribeirão Preto, University of São Paulo, São Paulo, Brazil; 3 Neuroscience and Behavior, Medical School of Ribeirão Preto, University of São Paulo, São Paulo, Brazil; 4 Department of Morphology, Physiology and Pathology, School of Odontology of Ribeirão Preto, University of São Paulo, São Paulo, Brazil; University of Insubria, ITALY

## Abstract

Cannabidiol (CBD) is a major *Cannabis sativa* constituent, which does not cause the typical marijuana psychoactivity. However, it has been shown to be active in a numerous pharmacological assays, including mice tests for anxiety, obsessive-compulsive disorder, depression and schizophrenia. In human trials the doses of CBD needed to achieve effects in anxiety and schizophrenia are high. We report now the synthesis of 3 fluorinated CBD derivatives, one of which, 4'-F-CBD (HUF-101) (**1**), is considerably more potent than CBD in behavioral assays in mice predictive of anxiolytic, antidepressant, antipsychotic and anti-compulsive activity. Similar to CBD, the anti-compulsive effects of HUF-101 depend on cannabinoid receptors.

## Introduction

Cannabidiol (CBD) is a major cannabinoid present in *Cannabis sativa*, which does not cause the typical effects of the psychoactive component, Δ^9^-tetrahydrocannabinol (THC). CBD was isolated from marijuana in 1940 by Adams *et al* [1] in the US and from Egyptian hashish by Jacob and Todd [2] in the UK. Its structure was elucidated in 1963 [3] and its absolute configuration was established in 1967 [4].

Numerous preclinical studies indicate that CBD exerts therapeutic effects in animal models of a wide range of health disorders, including neuropsychiatric conditions. Several mechanisms have been suggested to be involved in the actions of CBD: activation of TRPV1 channels, inhibition of uptake and metabolism of the endocannabinoid anandamide, inhibition of adenosine uptake, GPR55 antagonism, PPARγ and 5-HT1A receptors agonism, intracellular Ca^++^ increase, anti-oxidative effects etc. For reviews see [5–9]. More recently, a direct interaction with the mTOR/p70S6 kinase signaling has been proposed as a possible mechanism of CBD antipsychotic effects [10].

CBD was initially reported in 1980 to be anti-epileptic in a small trial of adult patients [11] and today plant extracts with high levels of CBD are widely administered to epileptic children. Positive results of larger clinical pediatric studies have already been published [12,13] and the drug has recently received orphan designation by regulatory agencies [14].

In the psychiatry field the initial findings that CBD attenuates the psychotomimetic and anxiogenic effects induced by high doses of THC in humans led to the assumption that this drug could possess antipsychotic and anxiolityc properties [15]. CBD indeed decreases behavioral changes induced in rodents by dopamine agonists or glutamate NMDA receptor antagonists [16–19]. Anxiolytic-like effects of CBD in rodents have also been described in different animal models after either acute or repeated administration [20–22]. CBD, at single doses of 300–600 mg/day, was also shown to attenuate public speaking-induced anxiety in healthy subjects and patients with social anxiety [23].

Extending these findings, we recently reported that CBD could also induce antidepressant- and anticompulsive-like effects in rodents tested in the forced swimming test and marble burying tests, respectively [24, 25].

Clinical antipsychotic effects of CBD (with doses reaching above 1 g/day) were initially described in open-label studies by Zuardi et al [26,27]. and have been recently confirmed (again, with doses of 800 mg/day) by Leweke et al. in a randomized, double blind clinical trial [28].

It is somewhat surprising that in spite of the very promising pharmacological/clinical effects of CBD and its lack of toxicity it has not been developed as a single drug. CBD is marketed together with Δ^9^-THC (in a 1:1 ratio) by GW Pharmaceuticals as Sativex, sold in Canada and several European countries for spasticity, due to multiple sclerosis [29]. One of the reasons may be the very high doses needed for activity in humans (see above). Therefore, the development of more potent CBD-type compounds is desirable.

The activity of numerous endogenous constituents and synthetic drugs has been enhanced by the introduction of a fluorine atom in their molecules [30,31]. Indeed, nearly 20% of new drugs reaching the pharmaceutical market contain a fluorine atom [31]. Many of these are lipophilic molecules such as steroids and fatty acid derivatives. The phytocannabinoids, including CBD, are likewise lipophilic. Hence we decided to fluorinate CBD at various positions and evaluate the activity of the novel molecules.

We report now the synthesis of 3 fluorinated CBD derivatives—one with the fluorine on the aromatic ring (HUF-101) (**1**), a second one with fluorine on the propylidene moiety of CBD diacetate (HUF-102) (**2**) and a third one with fluorine on the C-7 methyl group of the terpene ring of 8,9- dihydro-CBD (HUF-103) (**3**). We report the effects of these new molecules in rodent models predictive of anxiolytic, antidepressant, antipsychotic and anticompulsive effects.

## Materials and Methods

### 2.1 Chemistry

Chemicals and solvents were purchased from Biolab LTD (Jerusalem, Israel), J.T.Baker (Center Valley, PA, USA), Sigma-Aldrich (Rehovot, Israel), Acros (Yehud, Israel), Alfa Aesar (Lancashire, UK), Merck (Darmstadt, Germany), and Penta (Prague, Czech Republic) and were used without further purification, excluding dry diethyl ether and dichloromethane, which were refluxed over sodium and phosphorous pentoxide, respectively, and freshly distilled prior to use.

^1^H-NMR spectra were obtained using a Bruker AMX 300 MHz apparatus using the deuterated chloroform (CDCl_3_, *δ* = 7.25 ppm) with tetramethylsilane (TMS) as internal standard. Optical rotations were measured on II polarimeter in a 2.00 dm cell and 25°C. Elemental analysis was performed using a Perkin-Elmer (Boston, MA, USA) 2400 series II analyzer at the Hebrew University Microanalysis Laboratory. Thin-layer chromatography (TLC) was run on silica gel 60F_254_ plates (Merck). Column chromatography was performed on silica gel 60 Å (Merck). Compounds were located using a UV lamp at 254 nm.

#### 2.1.1 Synthesis of 4'-fluoro-cannabidiol, HUF-101 (1) Fig 1

**Fig 1:**
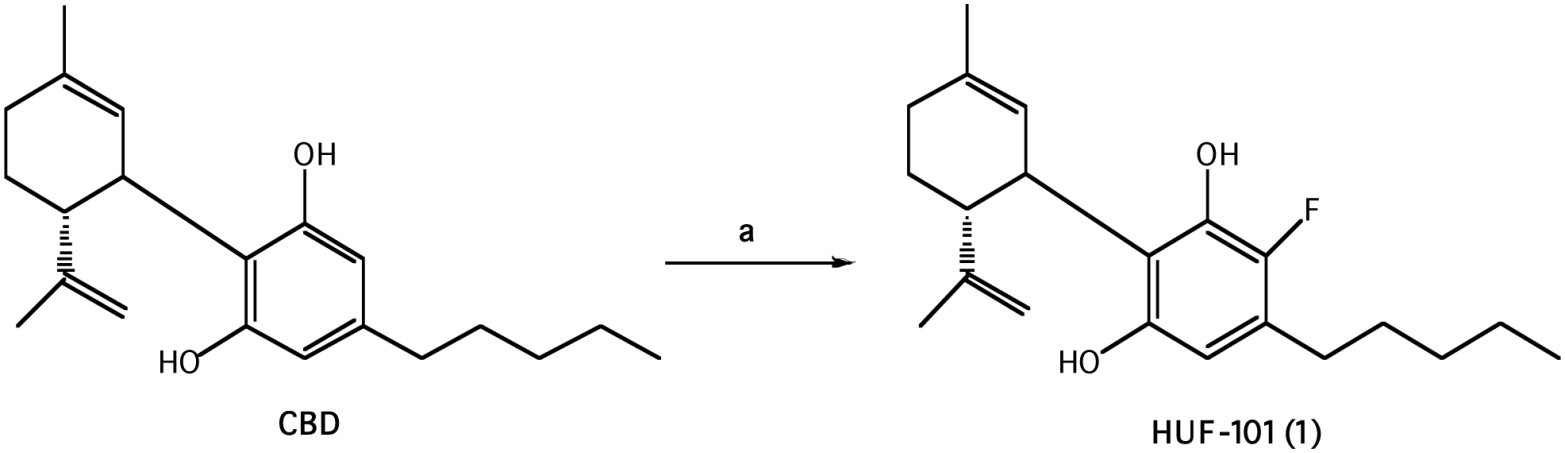
Reagents and conditions: (a) 1-fluoropyridinium triflate, CH_2_Cl_2_, r.t.

CBD was isolated from hashish following the procedure described by Gaoni and Mechoulam [32].

To a solution of CBD (942 mg, 3 mmol) in dry CH_2_Cl_2_ (42 ml) was added 1-fluoropyridinium triflate (742 mg, 3 mmol) and the reaction mixture was stirred at ambient temperature overnight. After dilution with CH_2_Cl_2_ the mixture was washed with saturated aqueous solution of NaHCO_3_. The organic layer was separated, dried over MgSO_4_ and evaporated. The oil obtained was chromatographed on a silica gel column (75 g). Elution with 2% ether in petroleum ether gave 4'-fluoro-cannabidiol (**1**) as a solid (300 mg, 27%) m.p. 59–61°C. ^1^H NMR (300 MHz, CDCl_3_) δ 6.17 (1H, s, Ar) 5.52 (1H, s), 4.56 (1H, s), 4.44 (1H, s), 3.92 (1H, s), 2.50 (2H, b), 2.19–2.05 (2H, b), 1.77 (3H, s), 0.86 (3H, t). MS, m/e = 332 (M^+^). Anal. Calcd for C_21_H_29_O_2_F: C, 75.86; H, 8.79; F,5.71. Found: C, 75.83; H, 8.92; F, 5.25.

#### 2.1.2 Synthesis of 10-fluoro-cannabidiol diacetate, HUF-102, (2) Fig 2

**Fig 2:**
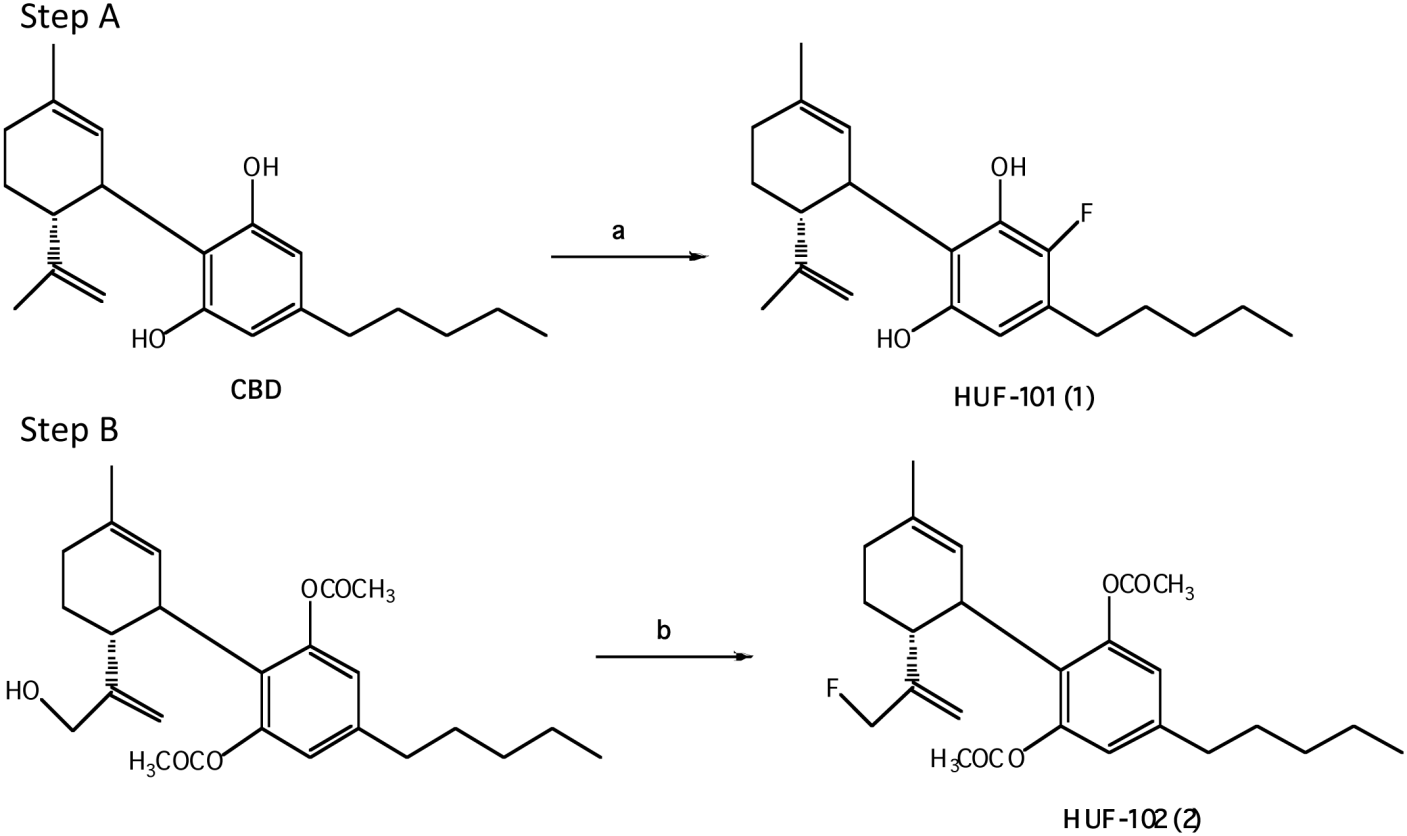
Reagents and conditions: (a) SeO_2_, t-BuOOH, CH_2_Cl_2_, r.t; (b) DAST, CH_2_Cl_2_, 0°C.

The known 10-hydroxy—cannabidiol—diacetate (**4)** (414 mg, 1 mmol), prepared following the published procedure [33], in dry CH_2_Cl_2_ (4 ml) was added under a N_2_ atmosphere to an ice-cold solution of DAST (0.18 ml, 1.5 mmol). After 15 min. at 0°C solid Na_2_CO_3_ (125 mg, 1 mmol) was added. The organic phase was then washed twice with cold 1 M aqueous Na_2_CO_3_ solution, followed by water. The organic layer was separated, dried over MgSO_4_, filtered and evaporated. The resulting crude material was purified on a silica gel column (20 g) using 10% ether in petroleum ether to provide the fluorinated product 10-fluoro-cannabidiol diacetate (**2**) (77.5 mg, 18.6%). ^1^H NMR (300 MHz, CDCl_3_) δ 6.74 (2H, s), 5.21 (1H, s), 5.01 (1H, s), 4.87 (1H, s), 4.60 (1H, s), 4.50 (1H, s), 3.6 (1H, b), 2.73 (1H, t), 2.57 (2H, t), 2.21 (6H, s), 2.08–1.59 (8H, ms), 1.32 (3H, s), 0.90 (3H, t). [α]^2^°_D_ = -55.3^ο^ in CHCl_3_, MS, m/e = 416 (M^+^). Analysis Calculated for C_25_H_33_O_4_F: C, 72.09; H, 7.99; F, 4.56. Found: C, 71.79; H, 7.93; F, 4.20.

#### 2.1.3 Synthesis of 8,9-dihydro-7-fluoro-CBD, HUF-103, (3) (Fig 3)

**Fig 3:**
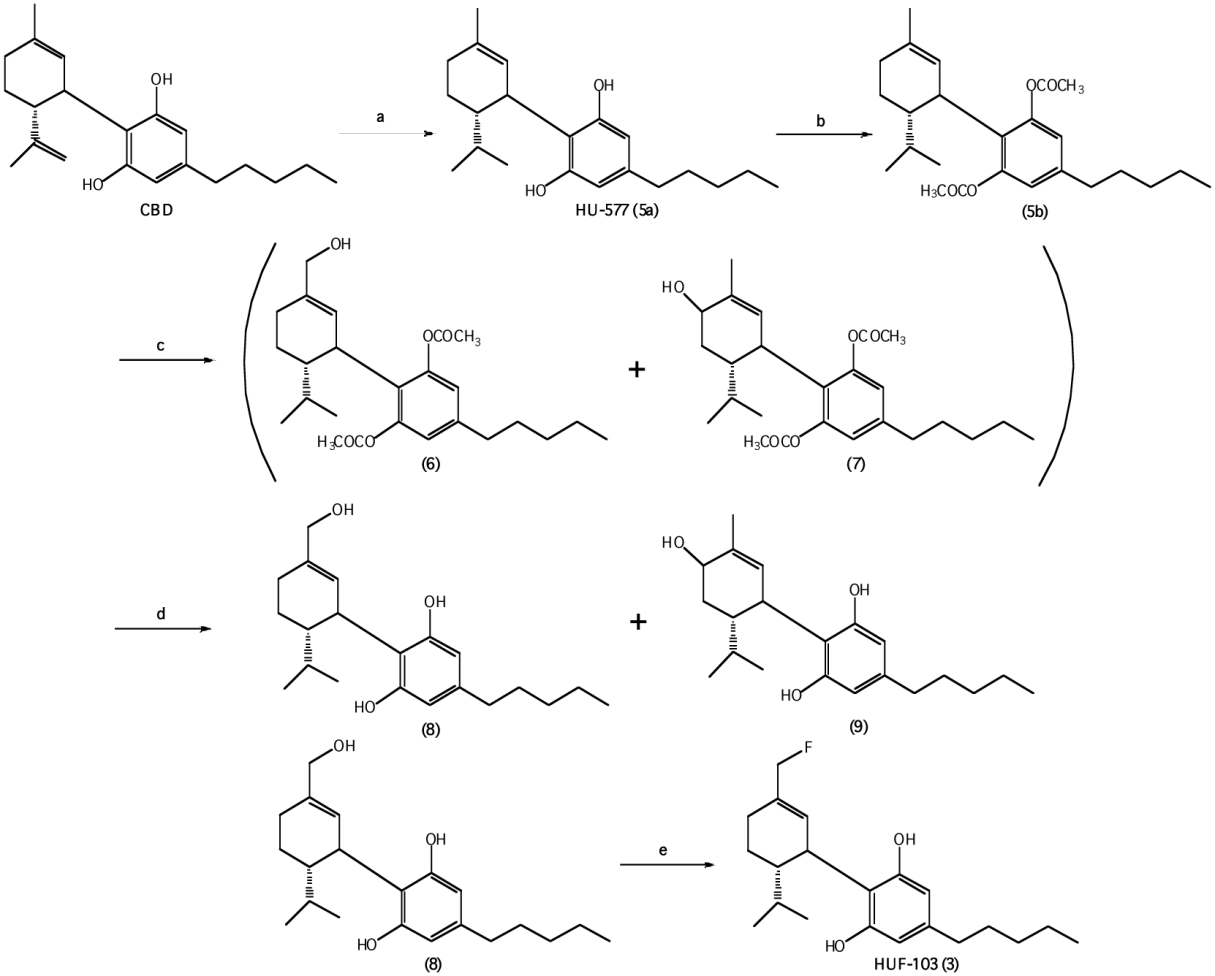
Reagents and conditions: (a) Pt(IV)oxide, H_2_, 10psi, EtOAc, r.t; (b) pyridine, acetic anhydride, r.t; (c) SeO_2_, EtOH, reflux; (d) NaBH_4_, EtOH, reflux; (e) DAST, CH_2_Cl_2_, -78°C–r.t.

CBD was isolated from hashish following the procedure described by Gaoni and Mechoulam [32]. The conversion of CBD into (-)-8,9-dihydro-7-hydroxy-CBD (**8**) has previously been reported by our group [33].

To a stirred solution of **8** (500 mg, 1.506 mmol) in CH_2_Cl_2_ (12 ml) at -78°C was added DAST (0.238 ml, 1.807 mmol, 1.2 equiv) at -78°C, stirred for 1 hr at the same temperature under N_2_ atmosphere, and the resulting mixture was slowly warmed to room temperature and stirred for 4 hrs monitoring by TLC. Solid Na_2_CO_3_ (160mg, 1.506 mmol) was added, the organic phase was washed with 1M aqueous Na_2_CO_3._ The solution was extracted with CH_2_Cl_2_. The combined extracts were washed with water and dried over MgSO_4_. After evaporation of the solvent under reduced pressure, the resulting residue was chromatographed using silica gel (1% ether-petroleum ether) to give **3** as light yellow oil (250 mg, 50%). ^1^HNMR (CDCl_3_, 300 MHz):*δ*6.200 (2H, s, Ar), 5.822 (1H, s, olefin), 4.752 (2H, d, CH_2_F), 3.962–3.923 (1H, m, benzyl), 2.567–2.484 (1H, td, *J* = 13.3, 2.7 Hz, allyl), 2.435–2.384 (2H, t, *J* = 7.5 Hz, benzyl), 1.882–1.734 (2H, m), 1.660 (6H, s. allyl CH_3_), 1.584–1.487 (2H, m), 1.285–1.248 (6H, m), 0.886–0.843 (3H, t, *J* = 6.3 Hz, terminal CH_3_); GC-MS m/z: 478 (silylation), 406, 334, 315, 299, 283. [α]^2^°_D_ = -55.3° in CHCl_3_, MS, m/e = 334 (M^+^). Analysis Calculated for C_21_H_31_FO_2_: C, 75.44; H, 9.28; F, 5.68. Found: C, 75.73; H, 9.52; F, 5.32.

### 2.2 Biological Assays

Male *Swiss* mice (30-45g) originated from the Central Animal Farm of the School of Medicine of Ribeirão Preto (FMRP-USP) were maintained in groups of five animals per box (41x33x17 cm) in a temperature controlled room (24±2°C) with a 12x12 h light-dark cycle. They received water and food *ad libitum* throughout the study period. The experimental protocols have been approved by the local Ethics Committee (CEUA, Medical School of Ribeirao Preto, Brazil, Protocol 058/2013) and are in accordance with the Brazilian and International regulations.

CBD (THC Pharm, 15–60 mg/kg), fluoxetine (Pharmaceutica, Brazil, 10 mg/kg), AM251 (Tocris, USA, 1 mg/kg) [34, 35], AM630 (Tocris, USA, 1 mg/kg) [35], **1** (1–10 mg/kg), **2** (1–60 mg/kg), **3** (1–30 mg/kg) and **5a** (1–30 mg/kg) were administered intraperitoneally (i.p.). All drugs but AM251 and AM630 were dissolved in 2% Tween 80 in sterile saline. The other two drugs were dissolved in 10% DMSO in sterile saline [34, 35].

#### 2.2.1 Behavioral tests

***2*.*2*.*1*.*1 Elevated plus-maze (EPM)*:** The wood-made EPM was located in a sound attenuated and temperature controlled room (23°C), with one incandescent light (40 W, 60 lux) placed 1.3 m away from the maze. The apparatus consisted of two opposing open arms (30 x 5 cm) perpendicular to two enclosed arms (30 x 5 x 40 cm), with a central platform common to all arms (5 x 5 cm). The apparatus was elevated 50 cm above the ground and an acrylic edge (1 cm) surrounded the open arms to prevent animal falls. In this model, anxiolytic drugs typically increase the exploration of the open arms without affecting the number of enclosed arms entries, which is usually used as a measure of general exploratory activity [36]. Thirty min after the drug injections the animals were placed on the central platform of the maze facing one of the enclosed arms. The test lasted for 5 min and the animal behavior was analyzed with the help of the Anymaze Software (version 4.5, Stoelting), which indicated the position of the animal in the maze and calculated the percentage of entries and time spent in the open arms and the number of entries in the enclosed arms. Animals were considered to enter an open or enclosed arm when 90% of their bodies were inside the region. All experiments were performed in the morning period (5 to 12 a.m.). ***2*.*2*.*1*.*2 Forced swimming test (FST)*:** Immediately after the EPM test the animals were submitted to 6 min of forced swimming in glass cylinders (height 25 cm, diameter 17 cm) containing 10 cm of water at 23–25°C. Immobility time (characterized by slow movements necessary to avoid drowning) was measured during the last 4-min period. The water was changed after each trial to prevent the influence of alarm substances [24]. During the tests no animal drowned or struggled to keep their heads above water. Therefore, there was no need for intervention by the experimenter. ***2*.*2*.*1*.*3 Prepulse inhibition (PPI)***: The PPI experiment was performed in independent groups of animals. The test was conducted simultaneously in two identical startle response systems (Med Associates, USA). A continuous acoustic signal provided a background white noise level of 65 dB. The pulse consists of a 105 dB white noise burst with a rise/decay of 5 ms and duration of 20 ms. The prepulse comprised pure 7000 Hz tones, 10 ms duration, with intensities set at 80, 85, and 90 dB. The setups were daily calibrated to ensure equal sensitivity throughout the experiments. Calibration was performed by adjusting the gain on the load cell amplifier to 150 arbitrary units (AU) at a standard weight appropriated for 40 g mice. The limits of the load cell were −2047 to +2047 AU. Thirty min after the injection of the tested compounds mice received an i.p. injection of amphetamine 10 mg/kg or vehicle. Animals were submitted to the PPI test 20 min after amphetamine or vehicle injection. After a 5 min acclimatization period in which the animal did not listen to any stimuli except the 65 dB background noise, mice were presented with a series of 10 stimuli (pulse alone). The first 10 pulse-alone trials allow for the within-session habituation to the startle stimulus and are not considered for PPI statistical analysis. The test consisted of 64 pseudo-random trials divided into eight different groups presented with an inter-stimulus interval of 30 s, and consisting of pulse alone (105 dB), prepulse alone (80, 85, or 90 dB), prepulse + pulse with 100 ms interval between prepulse and pulse, and no stimulus presented [18,19]. Prepulse stimulus did not elicit an acoustic startle response. Mean acoustic startle response to pulse-alone (P) trials and each prepulse + pulse (PP + P) trial was recorded for each subject. PPI was calculated by expressing the prepulse + pulse startle amplitude as a percentage of decrease from pulse-alone startle amplitude, according to the following formula: %PPI = 100–[100 × (PP + P/P)]. This transformation reduces statistical variability attributable to differences between animals and it is a direct PPI measure [18,19]. ***2*.*2*.*1*.*4 Marble burying test (MBT)*:** Independent groups of animals were submitted to the MBT. For this test twenty-five green clear glass marbles used were evenly spaced over the 5 cm sawdust layer-covered floor of a squared box (38 x 32 x 28 cm). Thirty minutes before the test the animals were pre-exposed for 5 min to the box. They are then placed in the center of marble-containing box. Thirty minutes later the number of buried marbles was recorded following the criteria for buried marbles proposed by Njung'e and Handley [37], namely that at least two-thirds were under sawdust.

#### 2.2.2 Statistical Analysis

Results were analyzed by one-way or two-way ANOVAs. The Duncan test was used for posthoc analysis. Significant level was set at p<0.05.

## Results

### 3.1 Chemistry

The direct fluorination of CBD on the aromatic ring was done with the electrophylic fluorinating agent 1-fluoropyridinium trifluoromethane sulfonate (known as 1-fluoropyridinium triflate) [38], to yield **1** (Fig 1).

The synthesis of the allylic fluoro derivative **2** was done in 2 steps (Fig 2) First CBD diacetate was oxidized with selenium dioxide and t-butyl peroxide to the known 10-hydroxy-CBD diacetate (**4**) [39]. Then the allylic hydroxyl group was replaced with fluorine with the nucleophylic fluorinating reagent DAST (diethyl amino sulfur trifluoride) [30], which is widely used for the direct transformation of aliphatic hydroxyl groups to C-F groups, to yield **2**. The exact procedure followed the experimental details reported by Boukerb et al [40].

The third compound in this series, **3** was prepared as described in Fig 3. First the 8,9 double bond was selectively reduced, to yield the known 8,9-dihydro-CBD (HU-577) (**5a**), which after acetylation to **5b** was oxidized with selenium dioxide to a mixture of 8,9-dihydro-7-hydroxy-CBD-diacetate **(6**) and 8,9-dihydro-6-hydroxy-CBD-diacetate (**7**), which could not be separated with ease. Hence the mixture was directly reduced with NaBH_4_ to yield 8,9-dihydro-7-hydroxy-CBD (**8**) and 8,9-dihydro-6-hydroxy-CBD (**9**), which could now be easily separated. The reaction sequence of CBD to compounds **8** and **9** was recently reported by us [33]. Compound **8** was fluorinated with DAST to give the desired 8,9-dihydro-7-fluoro-CBD (**3**).

### 3.2 Biological Assays

A summary of our results can be seen in Table 1.

**Table 1:** Effective doses of the tested compound in each behavioral test.

	Elevated plus maze (active doses)	Forced swimming (active doses)	Prepulse inhibition (active doses)	Marble burying (active doses)
HUF-101	3 mg/kg	3 mg/kg	3–10 mg/kg	10 mg/kg
HUF-102	Not effective (1–10 mg/kg)	Not effective (1–10 mg/kg)	Not effective (10–60 mg/kg)	
HUF-103	10 mg/kg	3–10 mg/kg	Not effective (3–30 mg/kg)	
HU-577	3 mg/kg	Not effective (1–10 mg/kg)	Not effective (3–30 mg/kg)	
CBD	30 mg/kg*	30 mg/kg**	30–60 mg/kg ***	30–60 mg/kg****

* Campos et al.[22],

** Zanelati et al.[24],

***Pedrazzi et al.[19],

**** Casarotto et al. [25].

#### 3.2.1 Elevated plus maze (EPM) and forced swimming (FST) tests

The EPM is currently the most widely used animal model of anxiety. Initially proposed by Handley and Mithani [41], it is based on the conflict generated in rodents by their natural tendency to explore novel environments versus the innate fear of bright and elevated places [42]. Factorial analysis indicates that the number of entries into the enclosed arm can be used to measure locomotor activity, while the percentage of entries and time spent in the open arms reflects the anxiety level of the animal [36]. Typically, CBD and other cannabinoids induce bell-shaped dose-response curves in this model, decreasing anxiety at small doses and being ineffective (or sometimes anxiogenic) at higher doses [43,44].

The FST is also the most extensively used animal model to predict antidepressant activity [45]. In this test rodents are exposed to force swimming in a cylinder filled with water. After an initial period of active escape behavior, the animal become immobile. Antidepressant-like drugs typically decrease the immobility time [45,46]. An initial study from our group showed that systemic administration of CBD induces antidepressant-like effects in mice submitted to the FST without changing locomotor behavior [24]. The antidepressant-like effects of CBD have been recently confirmed [47]. The authors showed, using another animal model, that a single CBD injection decreased depressive-associated behaviors over a prolonged period of time.

**Fig 4:**
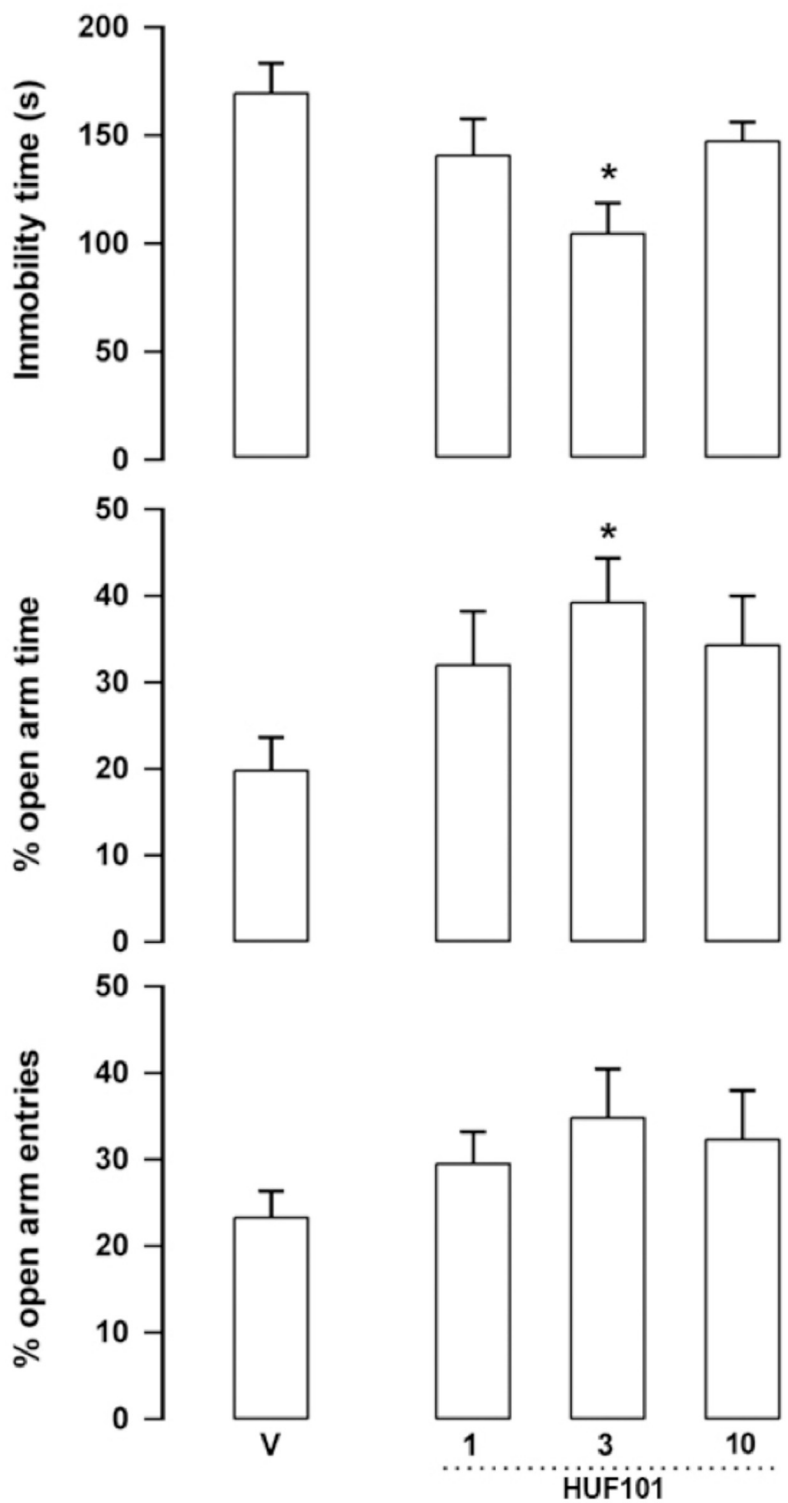
Effect of 1 (1, 3 and 10 mg/kg, n = 6, 8 and 6 animals, respectively) in Swiss male mice tested in the forced swim test (FST, upper panel) and elevated plus maze (EPM). Animals received an injection of vehicle (V, n = 7 animals/group) or **1** and were tested in the EPM 30min later. Immediately after the test they were submitted to the FST for 6 min. Data represents the means+SEM of immobility time in the FST and the % of open arm entries and time spent in these arms of the EPM. *indicates difference from V group (ANOVA followed by the Duncan test, p<0.05).

***HUF-101 (1)*:** At the dose of 3 mg/kg **1** decreased immobility time in the FST (F_3,23_ = 4.06, p = 0.019, Duncan, p<0.05) and the percentage of time spent in the open arms of the EPM (F_3,23_ = 2.89, p = 0.05, Duncan, p<0.05, (Fig 4). The drug did not change the number of enclosed arm entries (F_3,23_ = 0.25, NS, data not shown).

***HUF-102 (2)*:** The drug did not change open arm exploration (F_3,27_ = 1.97 and F_3,27_ = 1.15 for percentage of entries and time spent in the open arms, respectively, p>0.05) or the number of enclosed arm entries (F_3,27_ = 2.77, NS). No effect was found in immobility time in the FST (F_3,27_ = 0.45, NS). These data are not presented in a Fig.

**Fig 5:**
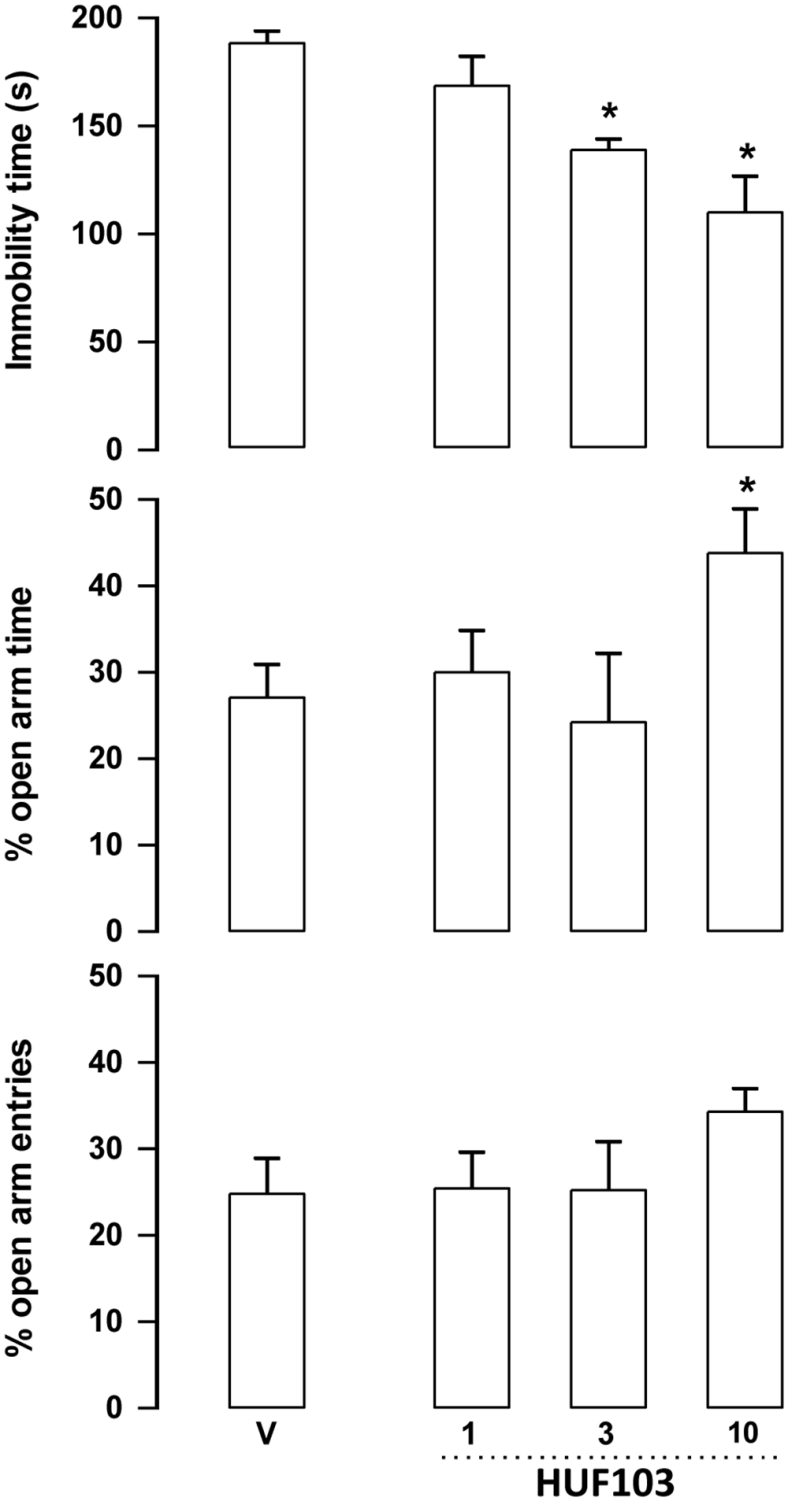
Effect of 3 (1, 3 and 10 mg/kg, n = 5, 5 and 6 animals, respectively) in Swiss male mice tested in the forced swim test (FST, upper panel) and elevated plus maze (EPM). *indicates difference from V group (ANOVA followed by the Duncan test, p<0.05). Further specification as in Fig 4.

***HUF-103 (3)*:** At the doses of 3 and 10 mg/kg **3** decreased immobility time in the FST (F_4,22_ = 5.35, p = 0.004, Duncan, p<0.05). The later dose also increased the percentage of time spent in the open arms of the EPM (F_3,19_ = 3.08, p = 0.052, Duncan, p<0.05) without changing the number of enclosed arm entries (Fig 5).

**Fig 6:**
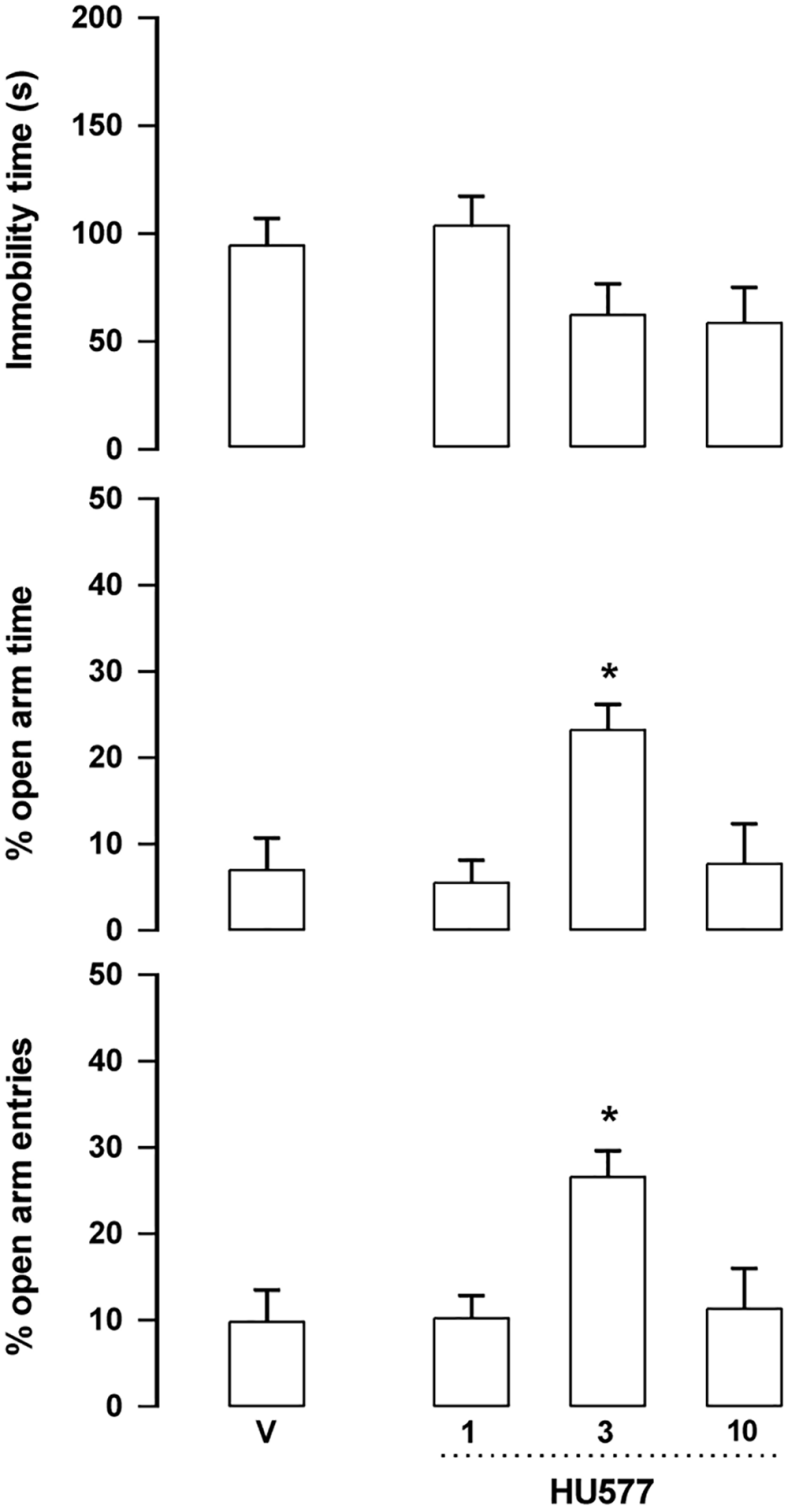
Effect of 5a (1, 3 and 10 mg/kg, n = 8–9 animals, respectively) in Swiss male mice tested in the forced swim test (FST, upper panel) and elevated plus maze (EPM). *indicates difference from V group (ANOVA followed by the Duncan test, p<0.05). Further specification as in Fig 4.

***HU-577 (5a)*:** The starting material for the synthesis of **1** and of **2** was CBD. However, the starting material for the synthesis of **3** was dihydro-CBD (**5a)**. Hence we also evaluated its activity. At the dose of 3 mg/kg **5a** increased the percentage of entries and time spent in the open arms entries (F_3,29_ = 5.42, p = 0.004 and F_3,29_ = 6.82, p = 0.001 respectively, Duncan, p<0.05). No change was observed in the number of enclosed arms. Although there was a trend, the drug also failed to change immobility time in the FST (F_3,29_ = 2.5, p = 0.079) (Fig 6).

#### 3.2.2 PPI (prepulse inhibition) test

PPI is a phenomenon that reflects the response attenuation induced by a weaker, non-startling stimulus (prepulse) that precedes a loud, startling acoustic stimulus (pulse) [48]. Schizophrenia patients often present a disruption of this normal inhibitory process [48]. Since psychotomimetic drugs such as amphetamine or glutamate NMDA receptor antagonists also disrupt this response in rodents and normal subjects, this model has been used to investigate the sensorimotor gating impairment found in these patients [49]. Acute or repeated CBD treatment attenuates the PPI impairment induced by dopamine agonists or NMDA receptor antagonists in rodents [18,19,50].

**Fig 7:**
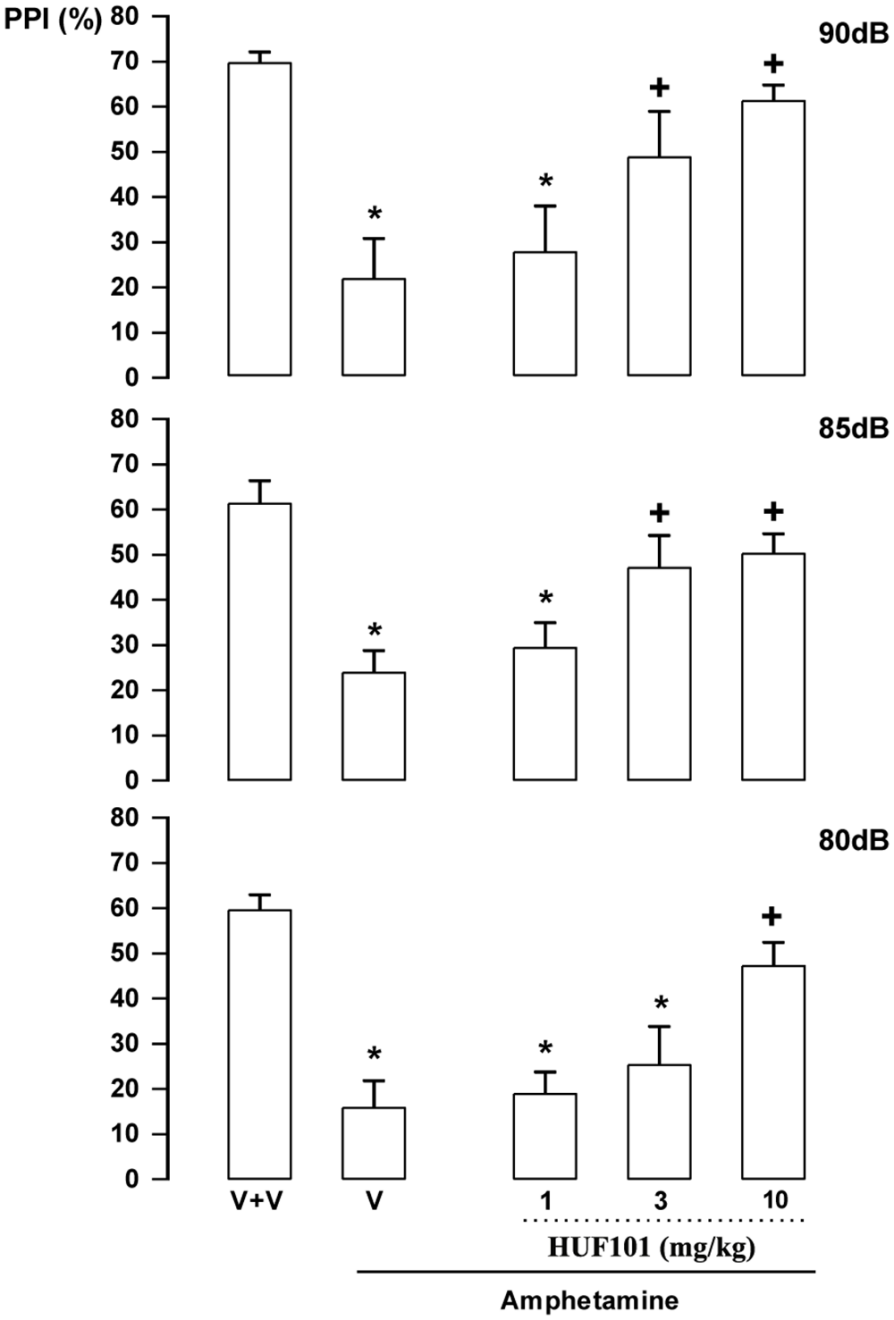
Effect of 1 (1, 3 and 10 mg/kg, n = 7, 6 and 15 animals, respectively) in Swiss male mice tested in the pre-pulse inhibition model. Animals received a first injection of vehicle (V) or **1** followed, 30 min later, by vehicle (n = 9) or amphetamine 10mg/kg (V+amphetamine group = 11 animals). *indicates difference from V+V group. + indicates difference from V+amphetamine group (ANOVA followed by the Duncan test, p<0.05).

***HUF-101 (1)*:** There were significant effects of intensity (F_2,86_ = 19.1, p<0.001); treatment (F_4,43_ = 11.4, p<0.001) and interaction between factors (F_8,86_ = 2.13, p = 0.041). Amphetamine impaired PPI in all intensities (Duncan, p<0.05). This effect was attenuated by **1** at 3 (85 and 90 dB) and 10 mg/kg (all intensities; Fig 7). By itself **1** (30 mg/kg) did not impair PPI (data not shown).

**Fig 8:**
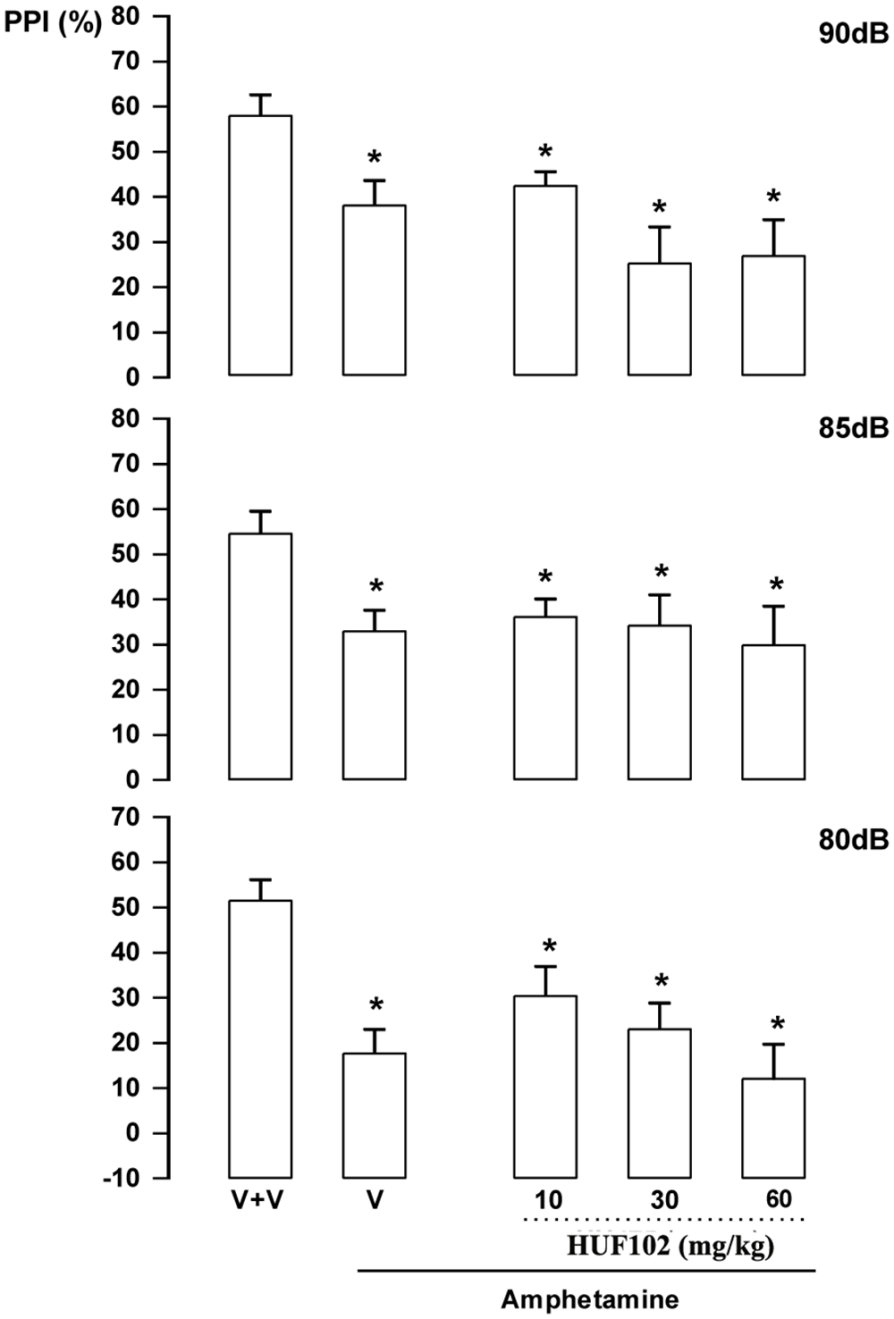
Lack of effect of 2 (10, 30 and 60 mg/kg, n = 7–8 animals/group) in Swiss male mice tested in the pre-pulse inhibition model. Animals received a first injection of vehicle (V) or 2 followed, 30 min later, by vehicle (n = 12) or amphetamine 10 mg/kg (V+amphetamine group = 11 animals). *indicates difference from V+V group (ANOVA followed by the Duncan test, p<0.05).

***HU-102 (2)*:** There were significant effects of intensity (F_2,92_ = 11.86, p<0.001) and treatment (F_5,46_ = 9.78, p<0.001) but no interaction between factors. Post hoc analysis showed that amphetamine impaired PPI in all intensities (Duncan, p<0.05). This effect was not attenuated by **2** at any dose (Fig 8). By itself **2** (60 mg/kg) did not impair PPI (data not shown).

**Fig 9:**
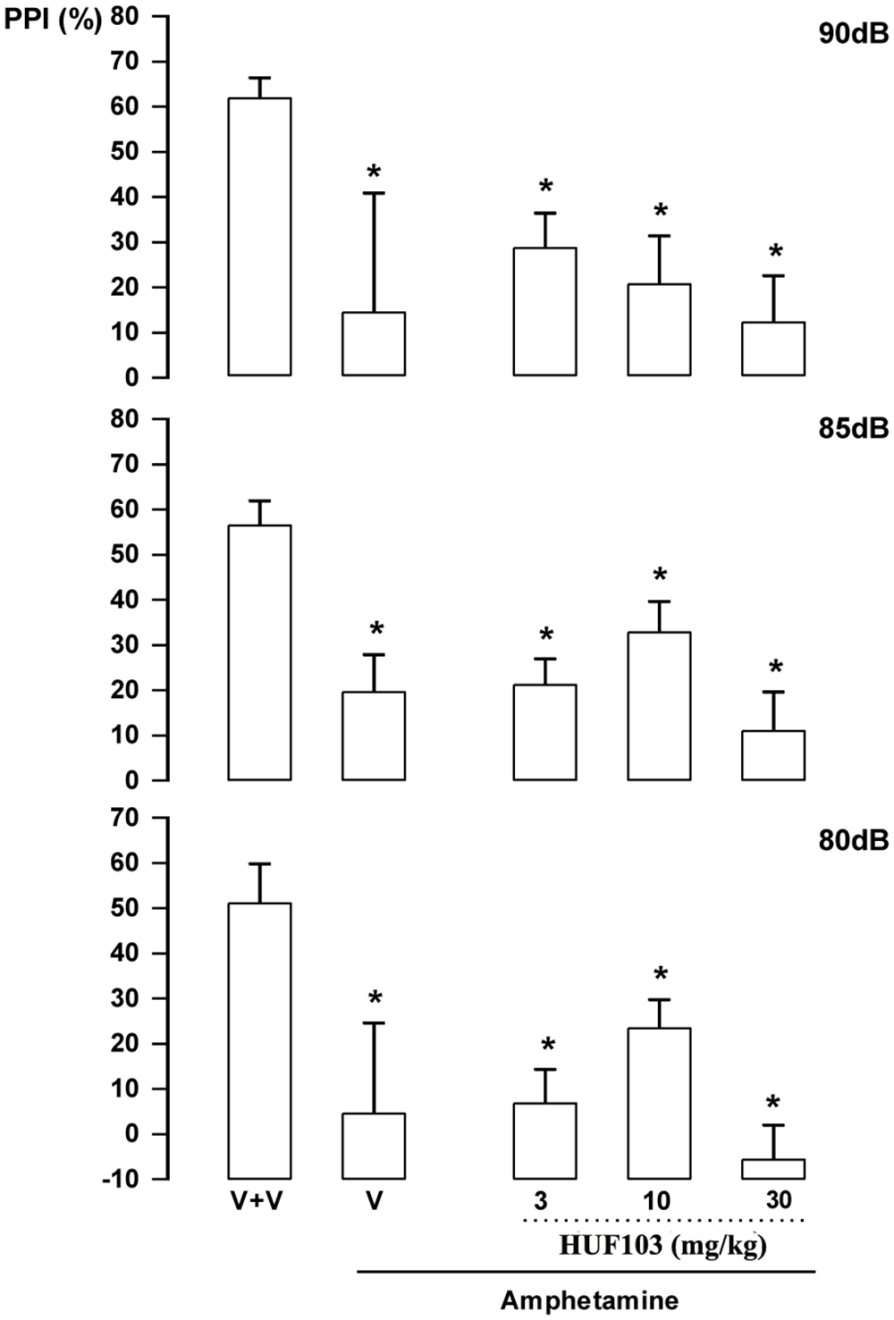
Lack of effect of 3 (10 and 30 mg/kg, n = 7–8 animals/group) in Swiss male mice tested in the pre-pulse inhibition model. Animals received a first injection of vehicle (V) or **3** followed, 30 min later, by vehicle (n = 7) or amphetamine 10 mg/kg (V+amphetamine group = 7 animals). Data represents the means+SEM. *indicates difference from V+V group (ANOVA followed by the Duncan test, p<0.05).

***HU-103 (3)*:** There were significant effects of intensity (F_2,72_ = 6.67, p = 0.002) and treatment (F_5,36_ = 8.53, p<0.001) but no interaction between factors (F_10,72_ = 0.80, NS). Post hoc analysis showed that amphetamine impaired PPI in all intensities (Duncan, p<0.05). This effect was not attenuated by **3** at any dose (Fig 9). By itself **3** at 30 mg/kg did not impair PPI (data not shown).

**Fig 10:**
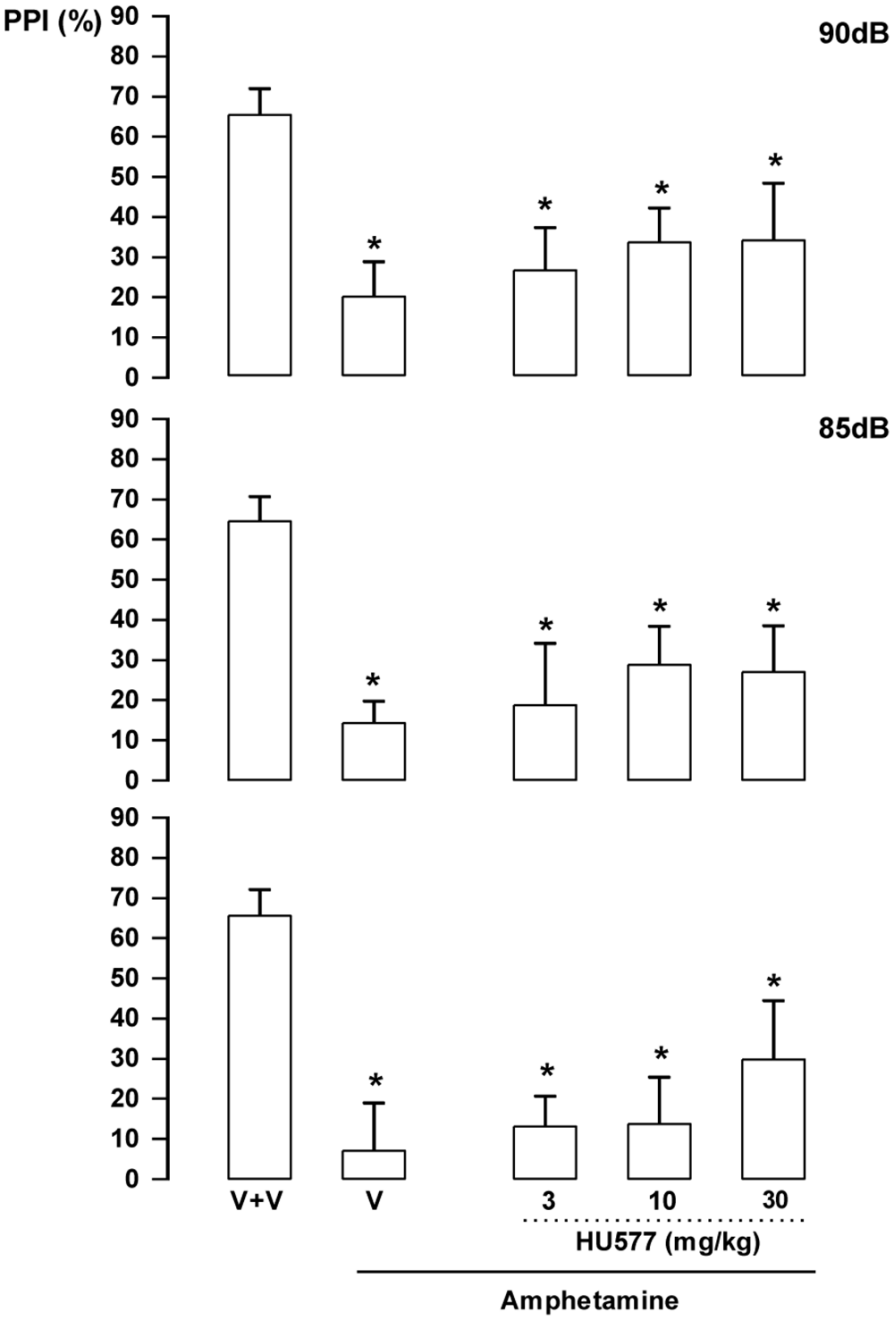
Lack of effect of 5a (3, 10 and 30 mg/kg, n = 8 animals/group) in Swiss male mice tested in the pre-pulse inhibition model. Animals received a first injection of vehicle (V) or **5a** followed, 30 min later, by vehicle or amphetamine 10 mg/kg (V+amphetamine). *indicates difference from V+V group (ANOVA followed by the Duncan test, p<0.05). Further specifications as in Fig 7.

***HU-577 (5a)*:** There were significant effects of treatment (F_5,42_ = 5.23, p = 0.001). Post hoc analysis showed that amphetamine impaired PPI in all intensities (p<0.05). This effect was not attenuated by **5a**, even when a higher dose (30 mg/kg) was used (Duncan, p>0.05; Fig 10). By itself, **5a** (30 mg/kg) did not change PPI response (data not shown).

#### 3.2.3 Startle response

No treatment modified the startle response to the pulse only stimulus.

#### 3.2.4 Marble burying test (MBT)

**Fig 11:**
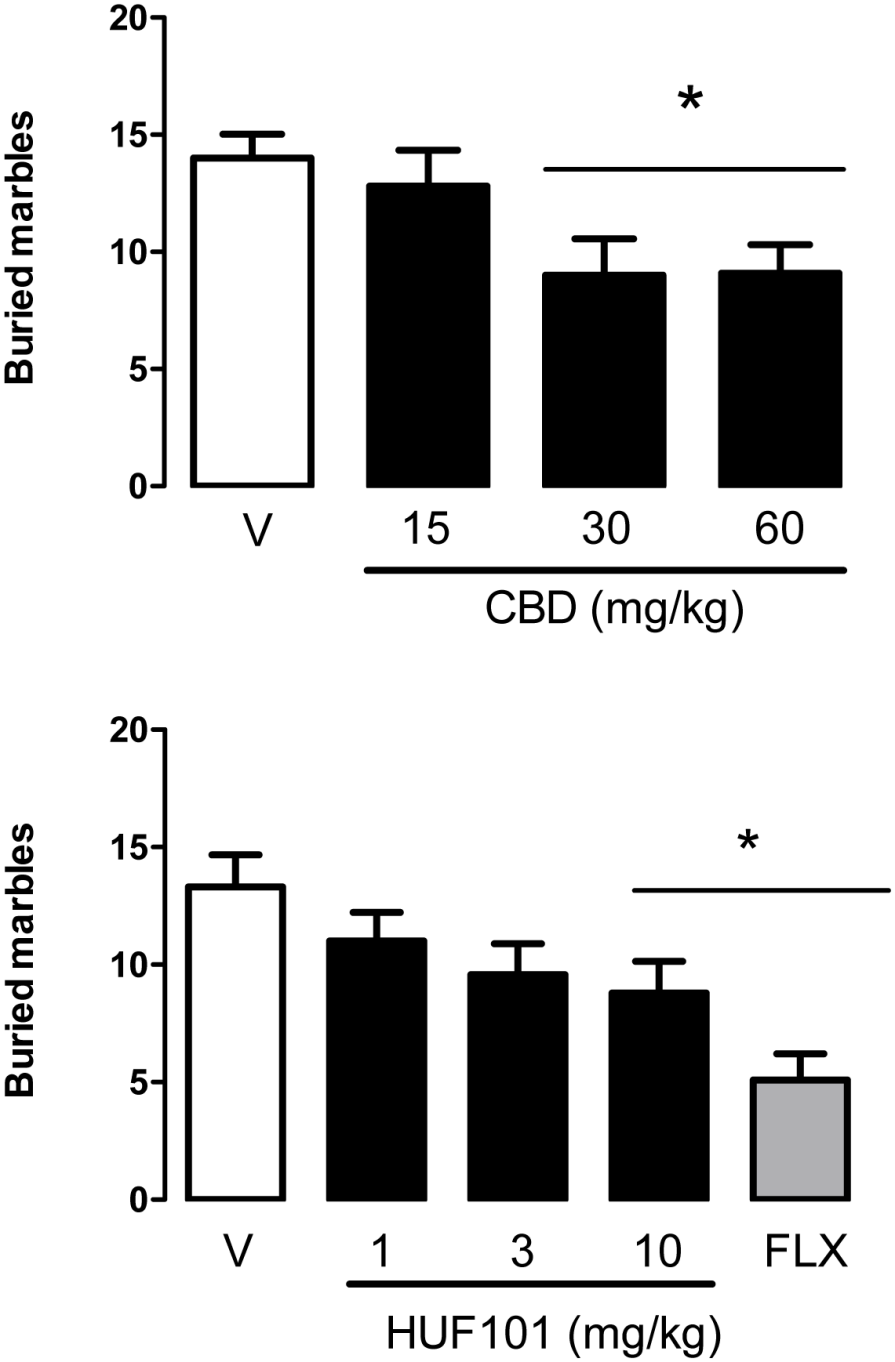
CBD (30–60 mg/kg, n = 10–11 animals/group), HUF-101 (10 mg/kg, n = 13–14 animals/group) and fluoxetine (10 mg/kg, n = 10 animals) decreased the number of buried marbles in the MBT test. *indicates difference from V+V group (ANOVA followed by the Duncan test, p<0.05).

**Fig 12:**
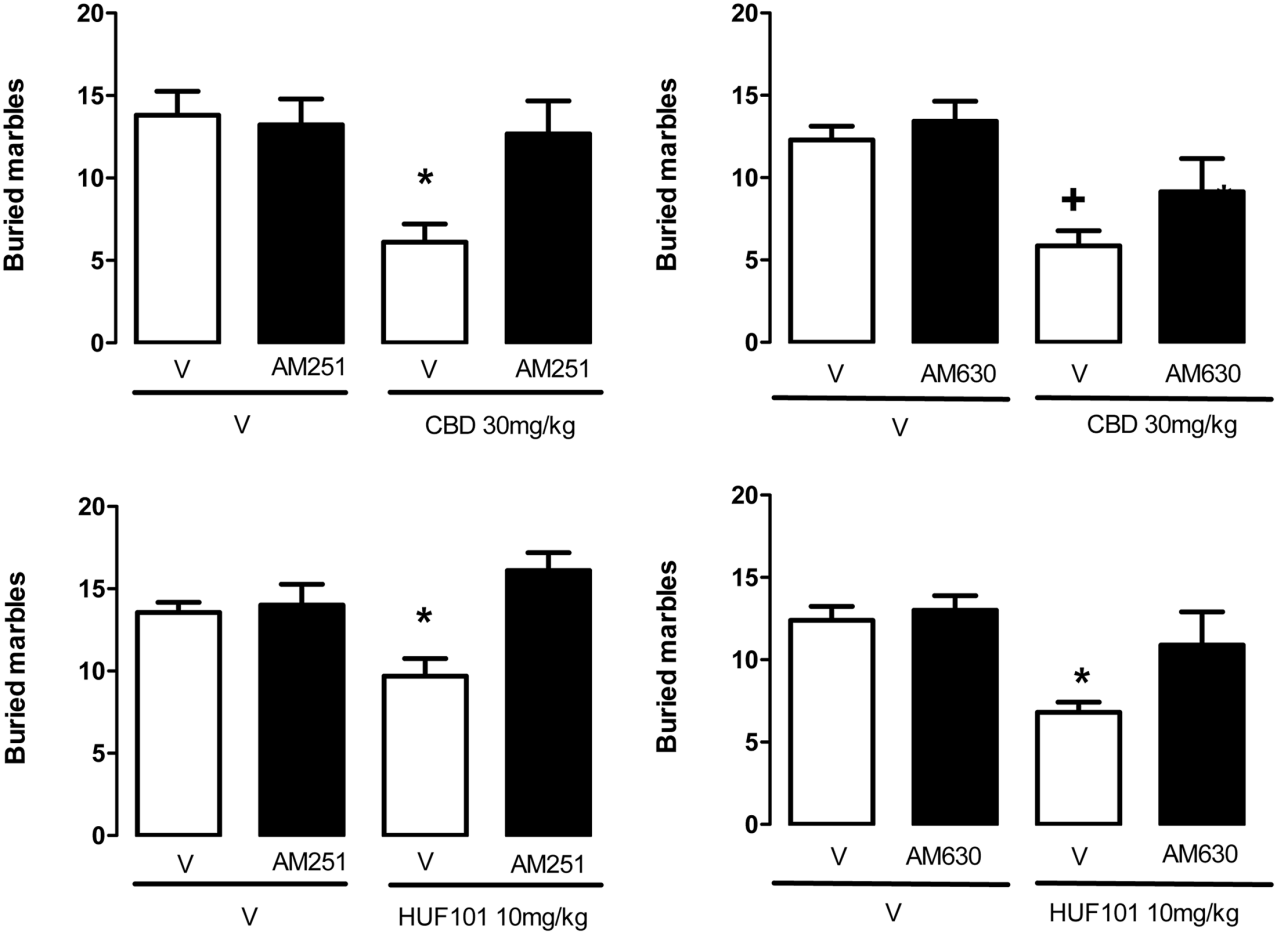
CBD (30 mg/kg, n = 7–9 animals/group) and HU-101 (10 mg/kg, n = 9–10 animals/group) decreased the number of buried marbles in the MBT. This effect was prevented by pre-treatment with the CB1 (AM251 1 mg/kg, n = 9 animals/group) or CB2 (AM630, 1 mg/kg, n = 7 animals/group) receptor antagonist. *indicates difference from all other groups. +indicates difference from V+V group (ANOVA followed by the Duncan test, p<0.05).

The MBT was initially proposed as an animal model of anxiety [37]. More recently it has been associated with a natural, repetitive behavior that can become compulsive [51]. Accordingly, it is sensitive to clinically effective anti-OCD drugs such as fluoxetine [51]. Replicating previous findings [25], CBD (30–60 mg/kg) decreased the number of buried marbles (F_3,39_ = 3.66, p = 0.02, Duncan, p<0.05, Fig 10). The same effect was observed after HUF-101 (10 mg/kg) or fluoxetine (F_5,51_ = 4.72, p = 0.002, Fig 11). The effects of CBD (30 mg/kg) and HUF-101 (10 mg/kg) were replicated in two additional experiments (CBD: F_1,33_ = 5.16, p = 0.03, F_1,24_ = 16.2, p<0.001; HUF-101: F_1,34_ = 7.94, p = 0.008, F_1,36_ = 9.97, Duncan, p<0.05, Fig 12). The effects of both drugs were prevented by pretreatment with the CB1 receptor antagonist AM251or the CB2 receptor antagonist AM630 (Duncan, p>0.05, Fig 12).

## Discussion

The EPM, FST, PPI and MBT are tests employed to unveil anxiolytic, antidepressant, antipsychotic and anticompulsive drug properties, respectively. All these properties have been shown to be associated with the CBD molecule (see above). In the present study we show that the fluorination of CBD leading to **1** enhances CBD potency in all these animal assays (Figs 4, 7, 11, 12 and Table 1). Compound **5a** was only effective in the EPM test (although there was a trend for an effect in the FST) (Fig 6). Compound **3** induced an effect, in the models for anxiety and depression, though with lower potency. However, the drug failed to attenuate PPI impairment induced by amphetamine, suggesting that it lacks antipsychotic-like effects (Fig 5). Compound **2** failed to cause any effect in the behavioral tests.

Although the knowledge of the molecular mechanisms responsible for CBD effects in the animal models used by us would have been an ideal starting point for the development of more potent compounds, our present poor understanding of these mechanisms prevents this approach. As mentioned in the introduction, numerous mechanisms have been suggested to be involved in the different actions of CBD [5–10]. Considering that these actions (e.g., anxiolytic, antidepressant, anticompulsive and antipsychotic) cannot clearly be explained by a single mechanism, to test all possibilities would be out of the scope of this paper. However, since we have previously found that CBD effects in the MBT, a single and reproductive test used to detect anticompulsive-like drugs effects, is prevented by pretreatment with a CB1 antagonist [25], we decided to test if a similar mechanism was involved in HUF-101 effects. This was, indeed, the case. Moreover, we expanded our previous results showing that a CB2 receptor antagonist also blocks CBD and HUF-101 effects in the MBT. CBD effects are probably indirect, since it has a low affinity for these receptors and is unable to produce the characteristic tetrad observed with high doses of CB1 agonists [8,16]. It can, however, inhibit the FAAH enzyme, responsible for anandamide metabolism [8]. This indirect activation of the endocannabinoid system has been proposed to explain several (but not all) effects of CBD, including facilitation of adult hippocampal neurogenesis [22], impairment of aversive memories [52] and anticompulsive [25].

Presumably the actions of the fluorinated CBD derivatives, particularly those of **1**, which parallel those of CBD, are based on the same mechanisms. The present findings in the MBT assay corroborate this proposition. However, this assumption clearly needs to be further investigated. Comparative pharmacokinetics studies are also required to determine if the greater potency of HUF-101 depends solely on pharmacodynamics changes induced by fluorination of the CBD molecule.

## Conclusion

We describe the synthesis of 3 fluorinated CBD derivatives **1**, **2** and **3**. Compound **1,** prepared by fluorination on the aromatic ring of CBD, is considerably more potent than CBD in behavioral assays in mice predictive of anxiolytic, antidepressant, antipsychotic and anticompulsive activity (Table 1). We also found that, similar to CBD, HUF-101 anticompulsive effects depend on CB1 and CB2 cannabinoid receptors. Fluorinated derivative **2**, in which the fluorine atom is on the propylidene entity (C-10) was not active (in doses of 1–10 mg/kg) in any of these assays, while **3,** in which the fluorine substitution is on the C-7 methyl group was less active than **1** in the anxiolytic and antidepressant assays and not active in the antipsychotic assay. CBD is already being evaluated as a therapeutic agent for these conditions, though at relatively high doses (see Introduction). In view of the higher potency of **1**, compared to CBD, this new CBD derivative may possibly be further developed as a therapeutic entity.
