# Correction: Fluorinated Cannabidiol Derivatives: Enhancement of Activity in Mice Models Predictive of Anxiolytic, Antidepressant and Antipsychotic Effects

**DOI:** 10.1371/journal.pone.0162087

**Published:** 2016-08-25

**Authors:** Aviva Breuer, Christeene G. Haj, Manoela V. Fogaça, Felipe V. Gomes, Nicole Rodrigues Silva, João Francisco Pedrazzi, Elaine A. Del Bel, Jaime C. Hallak, José A. Crippa, Antonio W. Zuardi, Raphael Mechoulam, Francisco S. Guimarães

The first step of Fig 2 appears erroneously in the publication as a repetition of Fig 1. The correct Fig 2 should show the oxidation of cannabidiol diacetate to 9-hydroxy-cannabidiol diacetate. Please see the correct Fig 2 here.

**Fig 2:**
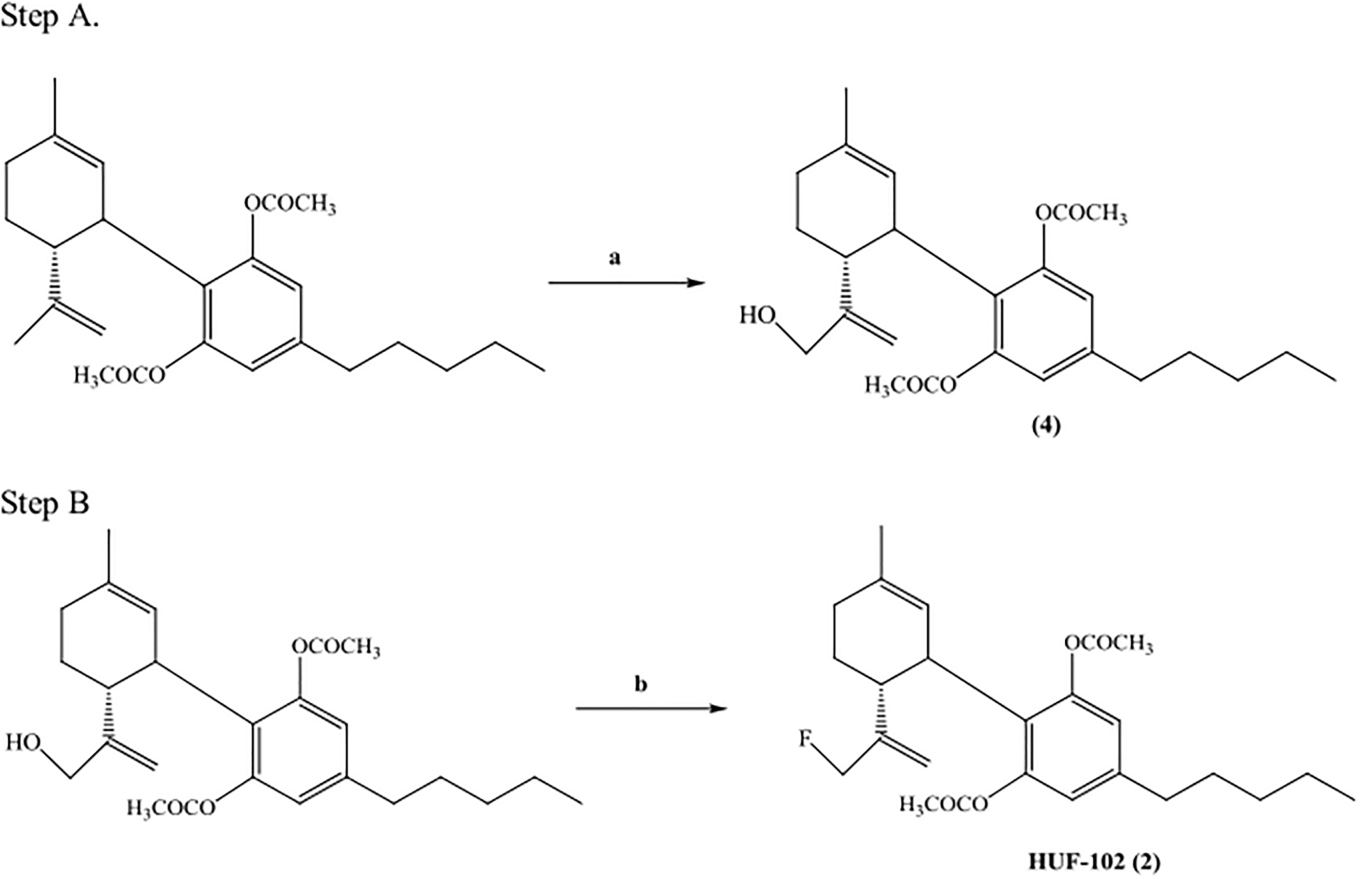
Synthesis of HUF-102(2). Reagents and conditions: (a) SeO_2_, t-BuOOH, CH_2_Cl_2_, r.t; (b) DAST, CH_2_Cl_2_, 0°C.
